# Substantially improved pharmacokinetics of recombinant human butyrylcholinesterase by fusion to human serum albumin

**DOI:** 10.1186/1472-6750-8-50

**Published:** 2008-05-16

**Authors:** Yue-Jin Huang, Paul M Lundy, Anthoula Lazaris, Yue Huang, Hernan Baldassarre, Bin Wang, Carl Turcotte, Mélanie Côté, Annie Bellemare, Annie S Bilodeau, Sandra Brouillard, Madjid Touati, Peter Herskovits, Isabelle Bégin, Nathalie Neveu, Eric Brochu, Janice Pierson, Duncan K Hockley, Douglas M Cerasoli, David E Lenz, Harvey Wilgus, Costas N Karatzas, Solomon Langermann

**Affiliations:** 1 PharmAthene Canada Inc. (formally Nexia Biotechnologies Inc.), 7150 Alexander-Fleming, Montreal, QC H4S 2C8, Canada; 2 Chemical Biological Defence Section, Defence Research and Development Canada-Suffield, Box 4000, Medicine Hat, AB T1B 8K6, Canada; 3 US Army Medical Research Institute of Chemical Defense, 3100 Ricketts Point Road, Aberdeen Proving Ground, MD 21010-5400, USA; 4 Quebec Transgenic Research Network, McGill University, 3655 Promenade Sir William Osler, Montreal, QC H3G 1Y6, Canada; 5 NewLife Fertility Centre, 4250 Sherwoodtowne Boulevard, Mississauga, ON L4Z 2G6, Canada; 6 CNKonsulting and Aegis Bioresearch, 251 Sherwood Road, Beaconsfield, QC H9W 2H4, Canada

## Abstract

**Background:**

Human butyrylcholinesterase (huBChE) has been shown to be an effective antidote against multiple LD_50 _of organophosphorus compounds. A prerequisite for such use of huBChE is a prolonged circulatory half-life. This study was undertaken to produce recombinant huBChE fused to human serum albumin (hSA) and characterize the fusion protein.

**Results:**

Secretion level of the fusion protein produced *in vitro *in BHK cells was ~30 mg/liter. Transgenic mice and goats generated with the fusion constructs expressed in their milk a bioactive protein at concentrations of 0.04–1.1 g/liter. BChE activity gel staining and a size exclusion chromatography (SEC)-HPLC revealed that the fusion protein consisted of predominant dimers and some monomers. The protein was confirmed to have expected molecular mass of ~150 kDa by Western blot. The purified fusion protein produced *in vitro *was injected intravenously into juvenile pigs for pharmacokinetic study. Analysis of a series of blood samples using the Ellman assay revealed a substantial enhancement of the plasma half-life of the fusion protein (~32 h) when compared with a transgenically produced huBChE preparation containing >70% tetramer (~3 h). *In vitro *nerve agent binding and inhibition experiments indicated that the fusion protein in the milk of transgenic mice had similar inhibition characteristics compared to human plasma BChE against the nerve agents tested.

**Conclusion:**

Both the pharmacokinetic study and the *in vitro *nerve agent binding and inhibition assay suggested that a fusion protein retaining both properties of huBChE and hSA is produced *in vitro *and *in vivo*. The production of the fusion protein in the milk of transgenic goats provided further evidence that sufficient quantities of BChE/hSA can be produced to serve as a cost-effective and reliable source of BChE for prophylaxis and post-exposure treatment.

## Background

Poisoning with organophosphorus (OP) compounds is a severe problem facing military personnel who may encounter lethal doses of these compounds in chemical warfare situations. Further, OP and other anticholinesterase agents are extensively used as pesticides and pose a substantial occupational and environmental risk. Human serum butyrylcholinesterase (huBChE, EC 3.1.1.8) is a globular, tetrameric molecule with a molecular mass of ~340 kDa [1]. Administration of exogenous huBChE has been shown to be an effective and safe alternative treatment for the prevention of OP nerve agent toxicity [2-7], although the physiological function of huBChE is largely elusive. In addition, it can hydrolyze many ester-containing drugs such as cocaine and succinylcholine [8]. Of the cholinesterases evaluated for nerve agent scavenging potential so far, huBChE purified from plasma has a relatively long half-life [9], a broad spectrum of activity against a variety of nerve agents including sarin, soman, tabun and VX, and limited, if any, physiological side effects [4]. However, huBChE may not be a viable option because it is only expressed at ~5 mg/liter in human plasma [10] and production of large quantities needed for civilian and military stockpiles is impractical. Recombinant huBChE has been expressed in bacteria [11] and in mammalian cell lines [12,13]. However, these recombinant production strategies have not yet produced sufficient quantities of functional BChE with a residence time similar to native huBChE to allow the economically reasonable development of recombinant enzyme as a prophylactic indication against OP poisoning. Recently it has been shown that large quantities of recombinant huBChE can be produced in the milk of transgenic goats for prophylaxis of humans at risk for exposure to OP nerve agents [14,15]. One way to achieve plasma stability and longer half-life of recombinant BChE while maintaining its biological activity is to provide a recombinantly produced BChE fused to human serum albumin (hSA).

It is well known that hSA, the most abundant protein in plasma, is a good carrier protein with a substantial circulatory half-life (up to 19 days in the human blood) and is widely distributed in vivo [16]. A number of proteins have been fused to albumin to improve their circulating half-lives and stability characteristics [17-19]. It has been reported that some therapeutic proteins genetically fused with hSA in general have longer circulating half-lives and improved pharmacokinetic and pharmacodynamic profiles compared to the non-fused proteins. Further, hSA has been shown to covalently bind OP agents *in vitro *[20,21] and *in vivo*, and therefore OP bound to hSA may serve as a biomarker of OP exposure in humans [22]. We hypothesized that it might be possible to delay the clearance of a rapidly cleared recombinant BChE via fusion to this slowly cleared plasma protein. This fusion protein may exhibit high plasma stability in the body and is expected to be either weakly or non-immunogenic for the organism in which it is used [23]. In addition, an affinity column, for example, an Affi-Gel Blue Gel column, can be used as an additional purification procedure for the hSA fused protein. Therefore, a series of DNA expression constructs harboring the sequence encoding the mature hSA fused in frame to the 3' end of the huBChE cDNA sequence with a DNA sequence encoding a (Gly)_6_-Ser amino acid linker were made. The fusion protein was produced first *in vitro *in BHK cells and used in a pharmacokinetic study as a proof-of-concept experiment, followed by fast generation of transgenic mice. Milk samples containing the fusion protein from a transgenic mouse were tested against a variety of nerve agents. Further, transgenic goats were generated for large-scale production of the fusion protein.

## Methods

### Construction of in vitro DNA expression vector CMV/BChE/hSA

Standard recombinant DNA methods employed herein have been described in detail [24]. All DNA cloning manipulations were performed using Escherichia coli Stbl2™ competent cells (Invitrogen, Burlington, ON, Canada). Restriction and modifying enzymes were purchased from New England BioLabs (Ipswich, MA). All chemicals used were reagent grade and purchased from Sigma-Aldrich (Oakville, ON, Canada), unless otherwise specified. Construct integrity was verified using DNA sequencing service provided by Mobix Lab (McMaster University, ON, Canada). Primers for sequencing and PCR were synthesized by Sigma-Genosys (Oakville, ON, Canada). PCR was performed using Ready-To-Go™ PCR beads (GE Healthcare Life Sciences, Baie d'Urfe, QC, Canada) or an Expand High Fidelity PCR kit (Roche Diagnostics, Laval, QC, Canada).

The huBChE cDNA was amplified by PCR from cDNA clone ATCC #65726 [25] (ATCC, Manassas, VA) with a sense primer Acb787 (5' AGA GAG GGG GCC CAA GAA GAT GAC ATC ATA ATT G 3') containing an ApaI site (underlined), partial Ig kappa signal sequence and an antisense primer Acb786 (5' CTG CGA GTT TAA ACT ATT AAT TAG AGA CCC ACA C 3') including a PmeI site (underlined) and partial 3' sequence of the huBChE cDNA. The PCR product was digested with ApaI and PmeI, purified using GFX matrix (GE Healthcare Life Sciences) and ligated into ApaI-PmeI digested CMV688M4 (kindly provided by Dr. J. F. Zhou, then Nexia Biotechnologies, Inc.) to generate CMV/BChE. PCR was performed using the CMV/BChE as a template with a sense primer Acb710 (5' GTG TAA CTC TCT TTG GAG AAA G 3') containing a portion of 5' BChE sequence and an antisense primer Acb853 (5' TAT AAG TTT AAA CAT ATA ATT ***GGA TCC *TCC ACC TCC GCC TCC **GAG ACC CAC ACA ACT TTC TTT CTT G 3') containing a PmeI site (underlined), a BamHI site (italic), a (Gly)_6_-Ser linker (bolded) followed by a portion of 3' BChE sequence. The PCR product was digested with XbaI, a restriction enzyme site located 185 bp downstream of the primer binding site of Acb710, and PmeI, and ligated to XbaI-PmeI digested CMV/BChE to generate an intermediate vector, CMV/BChEmd.

PCR was performed using Marathon-Ready™ human liver cDNA pool (Clontech, Mountain View, CA) as a template with a sense primer Acb854 (5' ATA TAA GGA TCC GAT GCA CAC AAG AGT GAG GTT GCT CAT C 3') containing a BamHI site (underlined) and partial sequence from the hSA cDNA 5' end (GenBank accession #V00495, without the signal sequence), and an antisense primer Acb855 (5' ATT TAA GTT TAA ACT CAT TAT AAG CCT AAG GCA GCT TGA CTT GC 3') including a PmeI site (underlined) and partial sequence from the hSA cDNA 3' end. This PCR product was digested with BamHI and PmeI, and inserted into BamHI-PmeI digested CMV/BChEmd to generate the final construct, CMV/BChE/hSA.

### Construction of in vivo DNA expression vectors

A DNA expression vector bCN/BChE [14] was digested with XhoI to remove the BChE insert. The BChE-less vector was blunt-ended by filling in with Klenow polymerase in the presence of dNTPs, and calf intestine alkaline phosphatase treated. The *in vitro *DNA expression vector CMV/BChE/hSA (see described above) was partially digested with NcoI to remove the BChE/hSA encoding sequences. The BChE/hSA fragment was blunt-ended by filling in with Klenow polymerase in the presence of dNTPs, and PmeI digested. The two blunt-ended fragments were ligated to generate bCN/BChE/hSA for pronuclear microinjection to produce transgenic mice. PCR cloning was performed to insert a neomycin fragment between the insulators and the β-casein promoter [14] in bCN/BChE/hSA to generate Neo-bCN/BChE/hSA for nuclear transfer in transgenic goats. For all the *in vivo *DNA expression vectors, the signal sequence is either from goat β-casein or BChE itself. Linear DNA (NotI digestion), free of bacterial sequences, was prepared [14]. DNA was quantified using a fluorometer and stored at 4°C.

### Transfection and selection of stable cell lines

BHK cells were transfected with Lipofectamine PLUS Reagent (Invitrogen) as per the manufacturer's recommendations using 4 μg of the CMV/BChE/hSA DNA. Briefly, the DNA was diluted to a final volume of 750 μl with DMEM (Invitrogen) and 20 μl of PLUS Reagent was added to the mixture. The Lipofectamine was diluted to a final volume of 750 μl with DMEM. After incubation at room temperature for 15 min, the Lipofectamine and DNA mixtures were combined and complexes were allowed to form for 15 min at room temperature. The lipid-DNA complex mixture was applied to BHK cells (ATCC), and the cells were allowed to incubate for 3 h at 37°C under 5% CO_2_. The cells were then cultured for another 24 h in fresh medium containing 20% fetal bovine serum (Invitrogen). Subsequently, stably transfected cells were selected in DMEM containing 10% fetal bovine serum and 300 μg/ml hygromycin B (Invitrogen). Colonies surviving selection were picked 7 to 14 days following transfection and expanded further.

The level of BChE activity in cell culture media from CMV/BChE/hSA transfected BHK cells was evaluated using a commercially available kit (Sigma-Aldrich). From over 100 clones tested, the one demonstrating the highest BChE activity (Clone 35) was further evaluated in roller bottles containing serum-free DMEM. A master cell bank was generated and used to initiate a hollow fiber bioreactor (CP2500 model, Biovest International, Worcester, MA) production run. Hollow fiber production of stable transfectants was established to produce the fusion protein.

### Production of founder and subsequent generation of transgenic animals

The production and maintenance of transgenic mice were conducted at the McIntyre Transgenic Core Facility of McGill University (Montreal, QC, Canada). Animal studies were carried out in accordance with Guidelines on the care and use of experimental animals from the Canadian Council of Animal Care (CCAC). Transgenic mice were generated essentially as described in [26] in a Friend virus B-type (FVB) background strain (Charles River Laboratories, Wilmington, MA). The bCN/BChE/hSA transgene was microinjected into the pronuclei of fertilized eggs, and 31 pups were born. At 2–3 weeks of age tail biopsies were taken, under anesthesia, and DNA was prepared according to standard procedures [24]. Transgenic founder mice were bred with wild-type mice of the same strain for the production of F1 and F2 generations.

The production and maintenance of transgenic goats were conducted at Caprine Production Farm (CPF) of then Nexia Biotechnologies Inc. Animal studies were carried out in accordance with Guidelines on the care and use of experimental animals from CCAC. The production of the founders and subsequent generation of transgenic goats was performed by nuclear transfer as described in Keefer *et al*. [27] using the DNA expression vector, Neo-bCN/BChE/hSA.

### PCR, Southern blot and FISH analysis

DNA samples from tails of mice and blood of goats were obtained 2–3 weeks and ~4 days after birth, respectively. Transgenic mice and goats were initially identified by PCR and subsequently confirmed by Southern blot analysis. PCR was performed by employing three sets of primers for each sample. Set A (Acb852, 5' CTT CCG TGG CCA GAA TGG ATG GGA GTG 3'/Acb883, 5' AAG AAA AGG AGT TCC GGG GC 3') amplified a 930 bp fragment from the 3' end of the BChE cDNA and hSA cDNA 5' end. Set B (Acb268, 5'AGG AGC ACA GTG CTC ATC CAG ATC 3'/Acb659, 5' GAC GCC CCA TCC TCA CTG ACT 3') amplified a 910 bp fragment of the insulator sequence. Set C for mice (Acb572, 5' TTC CTA GGA TGT GCT CCA GGC T 3'/Acb255, 5' GAA ACG GAA TGT TGT GGA GTG G 3') or goat (Acb256, 5' GAG GAA CAA CAG CAA ACA GAG 3'/Acb312, 5' ACC CTA CTG TCT TTC ATC AGC 3') amplified a 500 bp or a 360 bp portion of an endogenous mouse or goat β-casein gene, respectively, and ensured that the extracted DNA contained no inhibitors for the PCR reaction and it could therefore be amplified. Upon amplification the DNA samples were electrophoresed on a 1% agarose gel. Confirmation of intactness and copy number of the transgene was assessed with Southern blot analysis [28] using the DIG system (Roche Diagnostics). The transgene integration sites in the transgenic goats were investigated by FISH analysis with the protocol described elsewhere [28].

### Purification of BChE/hSA produced in vitro and in vivo

All the purification procedures were performed at 4°C unless otherwise noted. For BChE/hSA produced *in vitro *~30 liters of cell media were harvested from the hollow fiber system for purification. The media were brought to 50% saturation with solid ammonium sulfate, incubated for 1 h and centrifuged at 12,000 × g for 30 min. Supernatant was collected, brought to 80% saturation with ammonium sulfate and incubated for 1 h prior to centrifugation at 12,000 × g for 30 min. The pellet was resuspended in buffer A (20 mM sodium phosphate, pH 7.4, 1 mM EDTA) and dialyzed overnight against buffer B (buffer A + 100 mM NaCl). The dialysate was loaded onto a procainamide affinity column, synthesized in house and equilibrated in buffer B. The column was washed with 10 bed volumes of buffer C (buffer A + 150 mM NaCl), and eluted with buffer D (buffer A + 300 mM NaCl). Fractions were assayed for BChE activity and those with higher-than-background BChE activity were pooled. The pool was dialyzed overnight against buffer A and stored at 4°C. For BChE/hSA purification from the milk of transgenic goats, a similar methodology was developed based on precipitation of contaminating proteins with ammonium sulfate, followed by an additional HQ50 ion exchange chromatography (Applied BioSystems, Foster City, CA) prior to the procainamide affinity column chromatography [14]. The purity of the material was assessed by SDS-PAGE silver staining (Invitrogen) as described by the manufacturer.

For a BChE preparation with >70% tetramer, recombinant BChE produced from milk of transgenic goats was purified [14] and polyproline (Sigma-Aldrich) was added to the purified recombinant BChE at molar ratio of 2:1 to maximize tetramer formation. The mixture was incubated at room temperature for 2 h. Excess polyproline was removed by ultrafiltration with a 30 kDa cut-off nitrocellulose membrane. The percentage of tetramer was measured using (SEC)-HPLC [14].

### BChE activity assay (Ellman assay), non-denaturing BChE activity gel staining, Western blot analysis and SEC-HPLC

All the procedures were described in detail previously [14]. For SEC-HPLC protein size distribution was determined and compared with reference proteins including purified human plasma BChE (kindly provided by Dr. O. Lockridge, University of Nebraska, Omaha, NE). By using area counts, percentages of oligomeric forms of the fusion protein were determined.

### Pharmacokinetic study of in vitro purified BChE/hSA

Animals were used with Guidelines on the care and use of experimental animals from CCAC. All experimental protocols were approved by the institutional animal care committee. Purified BChE/hSA fusion protein produced *in vitro *was injected i.v. into 2 juvenile pigs of ~20 kg (10 mg/kg, using a converting equation of 720 units = 1 mg purified plasma huBChE [29]). As a control, a transgenically produced recombinant BChE preparation containing more than 70% tetramer was also injected i.v. into 4 juvenile pigs at a dose of 10 mg/kg. The pigs were anesthetized with isoflurane and 30% oxygen for placement of the catheters and prior to anesthetic delivery a control blood sample was drawn. Following anesthesia a second sample was taken and the plasma used for the control BChE activity. Following injection of the various samples, blood was taken at 1, 2.5, 5, 15, 30 min then at 1 h, 2 h, 4 and 8 h from the cannulated ear vein. After 8 h the animals were allowed to awaken and further samples were taken at 24, 48, 72 and 96 h (and in some cases beyond) from the external jugular vein. Plasma was produced from the drawn blood and analyzed using the Ellman assay. The data analysis was performed by MDS Pharma Services (Montreal, QC, Canada) using NONMEM^® ^version 5 (GloboMax, LLC, Ellicott City, MD) with a 2-compartment model.

### In vitro inhibition of BChE/hSA by OP agents

Various amounts of nerve agent solutions (2–2,000 nM) were added to milk samples collected from a F1 transgenic mouse. The samples containing the fusion protein were diluted with 0.1 M Tris-HCl (pH 8.0) at × 100 fold dilutions and incubated at 25°C for 5 min. Milk collected from a negative FVB mouse and treated in a similar way served as the negative control. Residual BChE activity (%) was measured by the Ellman assay at 25°C. Data were analyzed and plotted using Microsoft Excel (Microsoft, Redmond, WA).

## Results

### In vitro production and purification of BChE/hSA fusion protein

Production of the fusion protein from a selected clone, clone 35, using a hollow fiber system after transfection with the *in vitro *DNA expression vector, CMV/BChE/hSA, in BHK cells was initiated to generate sufficient material (~1 g in ~30 liters of media, 30 mg/liter) for purification with ammonium sulfate precipitation, followed by procainamide affinity chromatography. The purified fusion protein was analyzed by SDS-PAGE silver staining, indicating that the isolated material was ~50% pure (data not shown).

**Figure 1:**
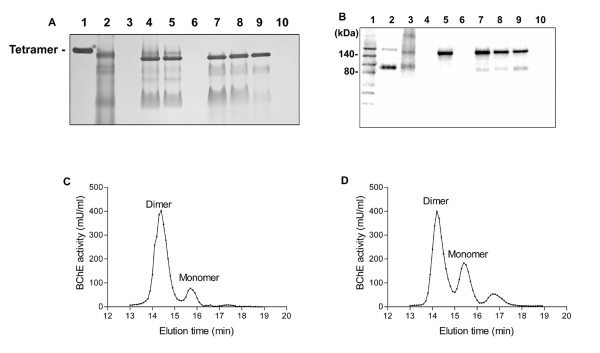
BChE/hSA fusion protein expression, activity and oligomeric forms. (A) BChE activity gel staining of samples from BChE/hSA produced *in vitro *and *in vivo*. Unless otherwise noted 15 μl (10 units/ml) of samples were loaded onto each lane of a precast 4–20% Tris-Glycine polyacrylamide non-denaturing gel in the following order: lane 1, purified plasma huBChE (tetramer indicated); lane 2, Harvest 8 of conditioned media of clone 35 from the hollow fiber system; lane 3, control conditioned media; lane 4, diluted milk sample from a F1 transgenic mouse, 307-1A7; lane 5, diluted milk sample from a F2 transgenic mouse, 307-1A7A4; lane 6, diluted milk sample from a nontransgenic FVB mouse (1:30); lane 7, diluted milk sample from a transgenic goat, 2237; lane 8, diluted milk sample from a transgenic goat, 2177; lane 9, purified BChE/hSA sample from milk of the goat 2177; lane 10, diluted milk sample from a nontransgenic goat (1:30). (B) Western blot analysis under denaturing and reducing conditions. Immunodetection was performed with a polyclonal anti-huBChE antibody (Dako, Mississauga, ON, Canada). Unless otherwise noted 15 μl (10 units/ml) of samples were loaded onto each lane of a precast 4–20% Tris-Glycine polyacrylamide gel in the following order: lane 1, Biotinylated molecular markers (Cell Signaling Technology, Inc., Danvers, MA); lane 2, purified plasma huBChE; lane 3, Harvest 8 of conditioned media of clone 35 from the hollow fiber system; lane 4, control conditioned media; lane 5, diluted milk sample from a F1 transgenic mouse, 307-1A7; lane 6, diluted milk sample from a nontransgenic FVB mouse (1:30); lane 7, diluted milk sample from a transgenic goat, 2237; lane 8, diluted milk sample from a transgenic goat, 2177; lane 9, purified BChE/hSA sample from milk of the goat 2177; lane 10, diluted milk sample from a nontransgenic goat (1:30). (C) SEC-HPLC analysis of milk from a transgenic mouse, 307-1A7A2. (D) SEC-HPLC analysis of milk from the transgenic goat, 2177.

An activity gel staining using conditioned media from BHK cells expressing the fusion protein demonstrated the secretion of an active enzyme, with the major band migrating faster than the plasma huBChE tetramer (Fig. 1A lane 2). Western blot analysis under denaturing and reducing conditions confirmed the expression of the fusion protein in BHK cells with expected molecular mass of ~150 kDa (Fig. 1B lane 3). A band appeared at ~90 kDa in the lane and it may belong to proteolytic products of the fusion protein. The BChE activity from the best producing clone was estimated at 0.7–0.75 units per million cells per 24 h.

### Pharmacokinetic study on BChE/hSA produced in vitro

**Figure 2:**
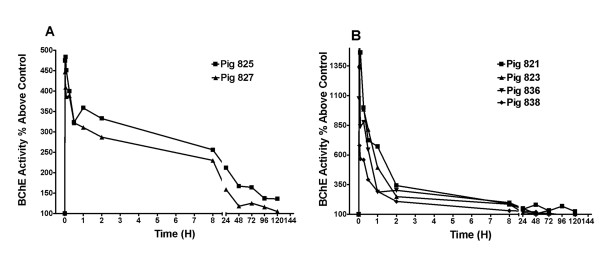
Clearance of BChE/hSA fusion protein produced *in vitro *(A) and a transgenically produced BChE preparation containing >70% tetramer (B) in juvenile pigs *in vivo*. Juvenile pigs were injected i.v. with the proteins and plasma BChE activity (%) above control (untreated pigs, ~1.6 unit/ml) was plotted versus time (hours) for each animal.

Plasma BChE activity in juvenile pigs following a single i.v. injection of 10 mg/kg BChE/hSA, produced *in vitro *and partially purified, or 10 mg/kg tetramer-enriched BChE, produced *in vivo *and purified, was analyzed using the Ellman assay with background BChE activity subtracted (Fig. 2A and 2B). The BChE activity curves of the fusion protein followed multi-exponential decay. However, the curves of tetramer-enriched BChE did not follow the multi-exponential decay; instead they appeared to have a more rapid rate of decline from the peak until ~200% above the control activity. Pharmacokinetic parameters derived from 2-compartmental analysis of the i.v. groups are summarized in Table 1. The results clearly revealed a substantial enhancement of the plasma half-life of the fusion protein (~32 h) when compared with the recombinant BChE transgenically produced in goat milk and enriched to contain more than 70% tetramer (~3 h) (Table 1; Fig. 2).

**Table 1:** Summary of the average (CV%) pharmacokinetic parameters from post-hoc bayesian estimates following protein i.v. injection in juvenile pigs

**Form of protein**	**n**	**K (1/h)**	**K12 (1/h)**	**K21 (1/h)**	**Vc (L)**	**Thalf (h)**	**Vehicle (unit/mL)**
BChE/hSA produced *in vitro*	2	0.0579 (0.0)	0.0993 (100)	0.103 (99.9)	0.581 (0.0)	32.4 (29.8)	1.83 (0.0)
BChE produced transgenically (>70% tetramer)	4	0.689 (28.4)	0.553 (18.4)	0.592 (0.000845)	0.120 (42.9)	2.74 (12.6)	1.45 (0.0)

CV%: The intersubject variation, reported as a percentage (%) of coefficient of variation;

K: The elimination rate constant from the central compartment;

K12: The rate constant of the protein from the central to the first peripheral compartment;

K21: The rate constant of the protein from the first peripheral to the central compartment;

Vc: The volume of the central compartment following i.v. administration;

Thalf: The apparent terminal elimination half-life;

Vehicle: The response detected by the analytical assay that is considered as background activity in untreated juvenile pig plasma, not related to the administration of the protein, using 720 units = 1 mg purified plasma huBChE and assay conditions as described in. [29].

### Identification of transgenic mice and expression of the fusion protein in the milk

Three F0 founder mice, two females and one male, were generated by pronuclear microinjection with the *in vivo *DNA expression vector, bCN/BChE/hSA. Southern blot analysis revealed that one transgenic female mouse carried ~40 copies of the transgene whereas the other two carried 1–2 copies in their genomes, respectively (Table 2). Two transgenic founders, the female with ~40 transgene copies and a male, bred with wild type mice of the same strain, transmitted the gene construct to their offspring. Upon breeding of the male line a Mendelian fashion of transmission was observed with ~50% of the F1 littermates found to be transgenic. When the female line was bred, a high level of transmission (~90%) was observed, indicating multiple integration sites of the transgene. Milk samples, collected from transgenic female mice, were analyzed using the Ellman assay, BChE activity gel staining, SEC-HPLC and Western blot. There were no detectable levels of BChE/hSA in the F1 females from the male line, probably due to the low copy number of the transgene integrated (data not shown). However, the results from the female line clearly indicated that the BChE activity was present in the milk of the founder and its female offspring with different transgene copies integrated (Table 2). BChE activity gel staining and analysis of the raw milk samples by SEC-HPLC revealed that the fusion protein consisted of a predominant form (~85%) corresponding to dimers in size and a minor monomer form (~13%) (Fig. 1A lanes 4 & 5; Fig. 1C). The fusion protein was confirmed to have the expected molecular mass of ~150 kDa when analyzed by Western blot under denaturing and reducing conditions (Fig. 1B lane 5).

**Table 2:** Summary of transgenic animals producing BChE/hSA fusion protein

**Transgenic animal**	**Animal ID**	**Generation**	**Sex**	**Transgene copy number**	**Integration site**	Expression in milk (g/liter)*
Mouse	307-1	F0	F	~40	ND	0.27
	307-1A2	F1	F	1–2	ND	0.40
	307-1A7	F1	F	8–10	ND	0.41
	307-1A7A2	F2	F	8–10	ND	0.24
	307-1A7A4	F2	F	1–2	ND	0.41

Goat	2176	F0	F	18–28	2	0.06
	2177	F0	F	6–10	1–2	1.10
	2178	F0	F	18–28	2–3	0.07
	2229	F0	F	10–14	1–2	0.07
	2231	F0	F	10–14	1–2	0.04
	2232	F0	F	10–14	1–2	0.09
	2233	F0	F	10–14	1–2	0.08
	2234	F0	F	10–14	1–2	0.35
	2236	F0	F	10–14	1–2	0.10
	2237	F0	F	10–14	1–2	0.23
	2238	F0	F	10–14	1–2	0.09
	2239	F0	F	10–14	1–2	0.11

*The concentrations for expression of BChE/hSA, estimated by densitometry where 720 units = 1 mg of purified plasma huBChE. [29], were the average of two independent assays. ND = not determined.

### Inhibition of BChE by OP nerve agents in vitro

**Figure 3:**
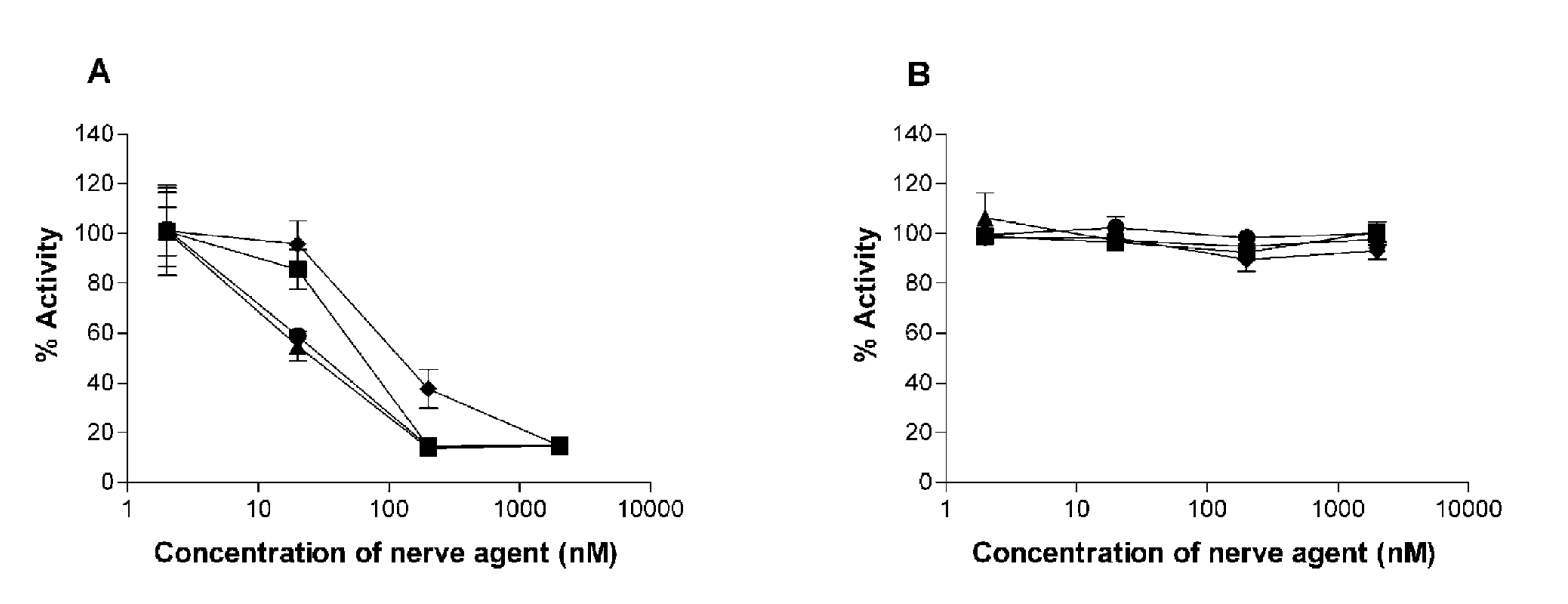
*In vitro *binding and inhibition of BChE/hSA fusion protein contained in the milk of transgenic mice by OP nerve agents. Binding and inhibition were performed with diluted raw mouse milk materials from a F1 transgenic mouse, 307-1A7 (A) and a control FVB mouse (B, 0.086 ± 0.03 unit/ml). The reactions were carried out in the presence of tabun (GA, ◆), sarin (GB, ■), soman (GD, ▲) and VX (●), respectively. Residual BChE activity was measured by the Ellman assay. Data points represent the mean ± SD from duplicates in each mouse.

Milk samples collected from a transgenic mouse and a nontransgenic mouse were incubated with various ratios of tabun (GA), sarin (GB), soman (GD) and VX in the nerve agent binding and inhibition assay (Fig. 3). The results indicated that the amount of the nerve agents required to inhibit 100% of enzyme activity (Fig. 3A) was in good agreement with that of plasma huBChE [4], as well as recombinant huBChE expressed in the milk of transgenic mice [14], suggesting that the fusion protein is similar to the plasma huBChE with comparable broad spectrum properties against a variety of nerve agents. Since the endogenous mouse BChE activity in the milk of non-transgenic mice is minimal [14], it is reasonable that no change of residual BChE activity in the non-transgenic milk was detected despite the addition of nerve agents with increased concentrations (Fig. 3B).

### Identification of transgenic goats and purification of the fusion protein in the milk

**Figure 4:**
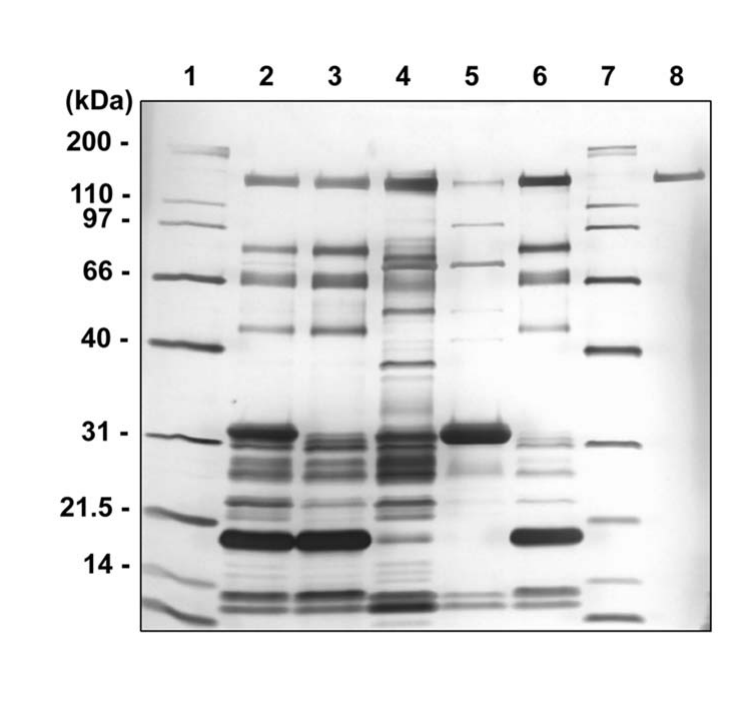
SDS-PAGE silver staining for purification of BChE/hSA fusion protein from the milk of the transgenic goat, 2177. 1 μg of samples were loaded onto each lane of a precast 4–20% Tris-Glycine polyacrylamide gel under denaturing and reducing conditions in the following order: lane 1, molecular weight marker (Bio-Rad, Mississauga, ON, Canada); lane 2, HQ 50 column load; lane 3, HQ50 column flow-through; lane 4, HQ50 column elution; lane 5, HQ50 column cleaning; lane 6, procainamide column flow-through; lane 7, molecular weight marker (Bio-Rad); lane 8, procainamide column elution.

A total of 19 female transgenic goats were generated using nuclear transfer in 3 primary goat fetal fibroblast cell lines transfected with the *in vivo *DNA expression vector, Neo-bCN/BChE/hSA, as determined by PCR, Southern blot and FISH analysis. 1–3 integration sites and a variety of transgene copies in their respective genomes were detected. While some of the clones manifested behavioral abnormalities and/or susceptibility to respiratory disease, most of these conditions improved dramatically or totally disappeared after one year of age [30]. Of 19 cloned transgenic goats 12 have been induced hormonally into lactation [31]. Goat 2177 expressed BChE/hSA in the milk in average ~1.1 g/liter whereas the others expressed at 0.04–0.3 g/liter, as determined by the Ellman assay (Table 2). Further, goat 2177 has entered into natural lactation, producing 1.1 g/liter of the fusion protein. ~7.5 g of bioactive BChE/hSA has been purified from ~8.7 liters of milk by a HQ 50 ion exchange chromatography, followed by a procainamide affinity chromatography (Fig. 4). The purity as assessed by SDS-PAGE silver staining is estimated to be >90% (Fig. 4 lane 8). Specific activity of the purified enzyme is ~400 units/mg. Non-denaturing polyacrylamide gels stained for BChE activity and SEC-HPLC analysis of the raw milk samples indicated that the fusion protein consisted of a major form (~60%) corresponding in size to dimers, a minor monomeric form (~28%) and some other minor components which may represent proteolytic products (Fig. 1A lanes 7–9; Fig 1D). Western blot under denaturing and reducing conditions revealed a protein with the expected molecular mass of ~150 kDa (Fig. 1B lanes 7–9). A minor band appeared at ~90 kDa in the lanes, suggesting proteolytic products of the fusion protein.

## Discussion

We have demonstrated that kilogram quantities of recombinant huBChE can be purified from the milk of transgenic goats where it is produced at concentrations of 1–5 g/liter and the recombinant BChE produced in the milk of transgenic goats was shown to be likely a useful bioscavenger of OP nerve agents [14,15]. The main objective of this study was to examine if the fusion of huBChE and hSA would act in a manner to yield a novel protein that retains the recombinant BChE's binding and inhibition against OP nerve agents and the slow clearance of hSA. During the course of the study different forms of recombinant BChE/hSA were produced. To prove the concept and due to availability of materials, partially purified BChE/hSA from BHK cells was used first in the pharmacokinetic study with the purified recombinant human BChE as a control. While the purity of the fusion protein may be a concern it is difficult to envision how impurities would affect the pharmacokinetics of the protein since only the active (and hence actual) material was measured. Then transgenic mice were generated. Non-purified milk samples were obtained from a transgenic mouse and used for the nerve agent *in vitro *binding and inhibition assay, as it is not practical to purify recombinant proteins from milk of transgenic mice because of small volume. When the nerve agent binding and inhibition experiment was performed the only available material was from transgenic mice. There was a few years' delay from producing the fusion protein from BHK cells to obtaining the protein from transgenic goats. Nevertheless, both the pharmacokinetic study and the *in vitro *nerve agent binding and inhibition assay, although using different forms of the fusion protein, suggested that a novel polypeptide retaining both properties of huBChE and hSA is produced *in vitro *and *in vivo*. The production of the fusion protein in the milk of transgenic goats provided further evidence that it is possible to produce such a protein in large scale and in a cost-effective way.

Given the fact that the recombinant human BChE expressed in the milk of transgenic goats is mainly in the dimer form [14], it is not surprised that the major eluted fusion protein peaks from the milk of transgenic mice and goats by SEC-HPLC are dimers in size. Dimers also appeared in other hSA-fused proteins although they were only present as minor components [32].

(Gly)_6_-Ser linker was engineered to fuse the hSA with the huBChE, facilitating the probability of independent folding as well as independence of action of the two portions of the fusion protein. Although its beneficial effects remain to be explored further, similar strategies have been successfully used in generation of other functional fusion proteins, for example, heavy and light chain Ig fusion proteins [33] and a recombinant hirudin genetically fused to albumin [34].

Other efforts to improve pharmacokinetic profiles of therapeutic proteins have included addition of dextran particles and polyethylene glycol (PEG) chains to the proteins. PEGylation often improves the pharmacokinetic and toxicity profiles of a recombinant protein and it may also reduce its immunogenicity [35]. However, high cost of PEGylation reagents and significantly reduced specific activity relative to some unmodified proteins [36,37] are among negative factors to be considered when PEGylation is assessed. The data presented in this study as well as from other hSA-fused therapeutic proteins suggest that the disadvantages of the PEGylation can be practically overcome by fusing a therapeutic protein to hSA genetically. Clinical trials of several fusion proteins, for example, hSA fused to human growth hormone, interleukin-2 and interferon alpha, all demonstrated safety, prolonged half-life, and biological activity [17-19].

It is well known that the mammary gland of transgenic animals is a good system for recombinant protein production [38]. A variety of recombinant human proteins, including immunoglobin, growth hormone and clotting factors, have been expressed and secreted in the milk of transgenic animals [39]. Expression of BChE/hSA in the milk of transgenic mice and goats represents just another good example. Since the expression level of BChE/hSA was assessed based on the BChE activity the actual secretion of the total BChE/hSA protein in the milk of transgenic animals should be at least doubled as half of the fusion protein is composed of the hSA. Based on a mathematically calculated model it has been suggested that the upper limit doses of 134, 115, and 249 mg/70 kg of human plasma derived BChE are sufficient to protect RBC AChE above 30% of baseline activity following a challenge with 1 × LD_50 _VX, soman, and sarin, respectively, thus preventing manifestation of severe toxic signs in human [40]. The predictions of huBChE doses were validated by *in vitro *experiments and data of published human studies. Therefore it is reasonable to predict that the upper limit doses described above for the plasma-derived huBChE may be doubled for the fusion protein to achieve the same protection. The experimental results presented in this study, along with other lines of evidence [14,38], indicate that transgenic production could provide high level expression of recombinant proteins in the milk of transgenic animals with flexible scale-up and significantly lower costs and risks compared to cell culture based production systems. This technique enables the large-scale production of complex or unique molecules that may not be produced efficiently by any other systems. The present study thus provides evidence to support the conclusion that in addition to the production of recombinant huBChE in the milk of transgenic goats [14], sufficient quantities of BChE/hSA can be produced from transgenic goats to serve as an effective and reliable source of BChE for prophylaxis and post-exposure treatment, alternatively to the purification of plasma huBChE.

## Conclusion

The pharmacokinetic study and the *in vitro *nerve agent binding and inhibition assay suggested that a huBChE/hSA fusion protein retaining both properties of huBChE and hSA is produced *in vitro *in BHK cells and *in vivo *in the milk of transgenic animals. It is possible to produce such a fusion protein in large scale and in a cost-effective way in the milk of transgenic goats.

## List of abbreviations used

BChE: butyrylcholinesterase; FVB: friend virus B-type; hSA: human serum albumin; huBChE: human butyrylcholinesterase; OP: organophosphorus compounds.

## Authors' contributions

YJH, AL, HB and CNK designed research; YJH, PML, AL, YH, HB, BW, CT, MC, AB, ASB, SB, MT, PH, IB, NN, EB, JP, DKH, DMC, DEL, HW and CNK performed research; YJH, PML, YH, HB, DMC, and CNK analyzed data; and YJH, CNK, and SL wrote the paper.
